# High frequency deep brain stimulation of the dorsal raphe nucleus prevents methamphetamine priming-induced reinstatement of drug seeking in rats

**DOI:** 10.1038/s41398-024-02895-y

**Published:** 2024-04-15

**Authors:** Libo Zhang, Shiqiu Meng, Enze Huang, Tianqi Di, Zengbo Ding, Shihao Huang, Wenjun Chen, Jiayi Zhang, Shenghong Zhao, Ting Yuwen, Yang Chen, Yanxue Xue, Feng Wang, Jie Shi, Yu Shi

**Affiliations:** 1 Shenzhen Key Laboratory for Drug Addiction and Medication Safety, Shenzhen Public Service Platform for Clinical Application of Medical Imaging, Department of Ultrasound, Peking University Shenzhen Hospital, Shenzhen-PKU-HKUST Medical Center, Shenzhen, China; 2 National Institute on Drug Dependence and Beijing Key Laboratory of Drug Dependence Research, Peking University, Beijing, China; 3 Henan Collaborative Innovation Center of Prevention and Treatment of Mental Disorder, the Second Affiliated Hospital of Xinxiang Medical University, Xinxiang, China

**Keywords:** Addiction, Neuroscience

## Abstract

Drug addiction represents a multifaceted and recurrent brain disorder that possesses the capability to create persistent and ineradicable pathological memory. Deep brain stimulation (DBS) has shown a therapeutic potential for neuropsychological disorders, while the precise stimulation targets and therapeutic parameters for addiction remain deficient. Among the crucial brain regions implicated in drug addiction, the dorsal raphe nucleus (DRN) has been found to exert an essential role in the manifestation of addiction memory. Thus, we investigated the effects of DRN DBS in the treatment of addiction and whether it might produce side effects by a series of behavioral assessments, including methamphetamine priming-induced reinstatement of drug seeking behaviors, food-induced conditioned place preference (CPP), open field test and elevated plus-maze test, and examined brain activity and connectivity after DBS of DRN. We found that high-frequency DBS of the DRN significantly lowered the CPP scores and the number of active-nosepokes in the methamphetamine-primed CPP test and the self-administration model. Moreover, both high-frequency and sham DBS group rats were able to establish significant food-induced place preference, and no significant difference was observed in the open field test and in the elevated plus-maze test between the two groups. Immunofluorescence staining and functional magnetic resonance imaging revealed that high-frequency DBS of the DRN could alter the activity and functional connectivity of brain regions related to addiction. These results indicate that high-frequency DBS of the DRN effectively inhibits methamphetamine priming-induced relapse and seeking behaviors in rats and provides a new target for the treatment of drug addiction.

## Introduction

Drug addiction is a major public health and social problem. According to the *World Drug Report 2023*, more than 39.5 million individuals were estimated to suffer from drug use disorders, in which the use of amphetamines suggested an overall increase during the last decade [1]. The rewarding and reinforcing properties of addictive drugs not only serve as essential factors in the progression towards addiction in individuals but are also linked with the conditioned stimulus, thereby contributing to the formation of associative memory [2]. As a kind of pathological memory, drug addiction engages aberrant adaptation of the reward system and neural network activity [3]. The enduring presence of this addiction memory, which is readily susceptible to activation, renders individuals with drug addiction highly prone to relapse.

Current treatment options for drug addiction, including medication and cognitive behavioral therapy, have limitations such as low efficacy, as well as tolerance and addiction caused by the therapeutic drugs. Consequently, it becomes imperative to explore avenues that ensure both safety and efficacy in clinical applications. Deep brain stimulation (DBS) has emerged as a valuable clinical treatment for addressing a diverse array of neuropsychiatric disorders [4, 5]. Research endeavors exploring the potential of DBS in addiction treatment have demonstrated that combining low-frequency DBS with dopamine D1 receptor antagonists in the nucleus accumbens (NAc) effectively suppresses drug-seeking behaviors in mice [6]. However, the application of DBS in addiction still encounters several challenges, including a limited selection of targets (mainly focusing on the NAc and subthalamic nucleus) and a lack of well-defined mechanisms [7, 8]. Therefore, the identification of novel therapeutic targets and a comprehensive understanding of the underlying neural mechanisms hold great significance for utilizing DBS as a treatment approach for drug addiction.

The dorsal raphe nucleus (DRN) is located in the midbrain and plays a crucial role in diverse functions and brain states, including anxiety [9], post-traumatic stress disorder [10, 11], depression [12, 13], sleep [14, 15], and social behaviors [16, 17]. Predominantly consisting of serotoninergic (5-HT) neurons, the DRN also comprises other neuron types, including dopaminergic, GABAergic (gamma-aminobutyric acid), glutamatergic, and peptidergic neurons [18]. Research have revealed that distinct subtypes of DRN neurons are involved in different natural rewards; for instance, DRN 5-HT neurons can be rapidly activated by stimuli related to appetite, while GABA neurons are suppressed by reward-related stimuli [19]. Moreover, a parallel reward pathway within the DRN is mediated by dopaminergic neurons [20]. Although the DRN is largely involved in reward-related behaviors, little is known about its role in methamphetamine-seeking behaviors. We therefore explored whether DRN can be a therapeutic target for mitigating methamphetamine relapse, along with identifying the appropriate stimulus parameters. Furthermore, we undertook an investigation into the impact of DRN DBS on the activities of brain regions associated with addiction by employing immunofluorescence staining and functional magnetic resonance imaging (fMRI) methodologies.

## Materials and methods

### Animals

Male Sprague-Dawley rats (260–280 g) were purchased from Beijing Vital River Laboratory Animal Technology Co., Ltd. and were given access to freely available food and water with a reverse 12/12 h light/dark cycle. All experimental procedures were performed in accordance with the National Institutes of Health’s Guide for the Care and Use of Laboratory Animals and were approved by the Biomedical Ethics Committee for Animal Use and Protection of Peking University.

### Implanting the stimulating electrodes and deep brain stimulation

As our previous study described [21], the rats were anesthetized with isoflurane and implanted with a stainless-steel bipolar electrode unilaterally into the DRN at the following coordinates: anterior/posterior, −7.5 mm; medial/lateral, 0 mm; and dorsal/ventral, −6.4 mm. After the surgery, the rats were housed individually for a 3–5-day recovery before behavioral experiments.

Monophasic square pulses were delivered to the DRN using a current-based stimulator through a cable connected to the implanted electrodes. The stimulation parameters were high-frequency (130 Hz), low-frequency (20 Hz) or sham (0 Hz) pulse frequencies, 150 μA pulse amplitude, and 100 μs pulse width. All placement sites were checked after behavioral tests, and electrodes implanted outside the DRN were excluded from the group.

### Conditioned place preference

The conditioned place preference (CPP) procedure was performed as described previously [21]. Baseline preference was assessed by placing the rats in the center chamber of the CPP apparatus and allowing them to explore all three chambers freely for 15 min, rats that showed a strong unconditioned preference for either side chamber (i.e., > 540 s) were excluded from the experiments. Then, (a) In the drug-induced CPP experiments, the rats were trained for eight consecutive days with alternating injections of methamphetamine (1 mg/kg, intraperitoneal injections, *i.p*.) or saline (1 ml/kg, *i.p*.) and were confined to the conditioning chambers for 45 min after each injection before being returned to their home cages; (b) In the food-induced CPP experiment, rats were trained for eight consecutive days with alternating exposure to the food (chocolate) following a 60 min DBS in the food-paired chamber or nothing in the opposite chamber and then were confined for 45 min before being returned to their home cage. The test for the expression of CPP was identical to the initial baseline preference assessment and was performed on the following day after training.

Then all rats were divided into sham, high-frequency, and low-frequency DBS groups in an unbiased random manner and underwent a free-access CPP extinction procedure. Similar to the expression test, the rats were allowed to move freely between compartments during each extinction session and a total of eight extinction sessions were performed until the rats showed no obvious preference for either chamber. On the last day, rats were subjected to DBS for 60 min, received an injection of methamphetamine (1 mg/kg) and were tested for preference. The time spent (in seconds) in the methamphetamine-paired chamber minus the time spent in the saline-paired chamber was calculated as the index of the CPP score.

### Intravenous methamphetamine self-administration

The self-administration procedure was based on previous studies [22]. Rats were implanted with the electrode and jugular catheters and underwent 3 days of recovery. Then, the rats were trained to self-administer methamphetamine (0.1 mg/kg/infusion) at the dark cycle with 3 h/d (three 1-h sessions were separated by 5 min) for 10 consecutive days in the operant chambers (AniLab Software and Instruments). Two nosepoke holes were located on the two sides 5 cm above the bottom of the chamber, in which the left nosepoke was the active operandum, while the right nosepoke was the inactive operandum. When the active operandum was triggered, an infusion of methamphetamine paired with a 5 s tone-light cue was given, while the trigger of inactive operandum led to no response. After each infusion, a 40 s timeout period was given, during which the nosepokes were recorded but no programmed consequences would be produced. The number of methamphetamine infusions was limited to 30 times per hour to prevent overdose.

Then the rats were divided into sham and high-frequency DBS groups and performed a free-access self-administration extinction procedure. The conditions were identical to those during training, with the exception that nosepoke responses led to the 5-s tone-light cue but not methamphetamine infusions. The rats underwent extinction training until their nosepokes on the active operandum were less than 20% of the mean nosepokes during the last 3 days of methamphetamine self-administration training for at least two consecutive days. On the test day, rats were subjected to DBS for 60 min and received an injection of methamphetamine (1 mg/kg). A 60 min test procedure was then performed on all rats. The testing conditions were identical to the training phase except that active nosepokes did not lead to methamphetamine infusions.

### Elevated plus maze test

The elevated plus maze comprised four arms arranged in a plus-shaped configuration. All rats received a 60-min DBS in their home cages, and then each rat was placed in the central zone of the elevated plus maze with its head facing an open arm. The rat was allowed to freely explore the elevated plus maze for 5 min.

### Open field test

The open field test apparatus consisted of a square arena that was 75 cm long, 75 cm wide, and 40 cm high. All rats received a 60-min DBS in their home cages, and then each individual rat was placed in the center of the arena and allowed to freely explore for 5 min.

### Immunofluorescence staining

The brains were dissected and post-fixed before frozen in liquid nitrogen. Coronal sections were cut at 25 mm thickness and incubated in phosphate buffered saline containing 0.5% Triton X-100 and 10% normal donkey serum for 1 h at 37 °C. The sections were then incubated with rabbit antibody to c-Fos (1:500, Cell Signaling Technology) for 24 h at 4 °C. After incubation with the primary antibodies, sections were rinsed with phosphate buffered saline four times for 5 min each and then incubated with secondary antibodies (Alexa Fluor 488-conjugated goat anti-rabbit, 1:500, Invitrogen) for 3 h at room temperature. After incubation with the secondary antibodies, the sections were rinsed four times for 5 min each, mounted, and coverslipped. The Fos+ cells were quantified in a blinded fashion by selecting at least three slices from each brain region of each rat and then measuring the number.

### Functional magnetic resonance imaging

fMRI data were acquired from a 7.0-T animal MRI scanner (PharmaScan 70/16 US, Bruker, Germany) with a coil for rats. Anesthesia was induced with 3% isoflurane in oxygen-enriched air. During scanning, isoflurane (1.5–2.0%) in oxygen-enriched air was delivered via a rat mask. Core body temperature, cardiac rates and respiration rates were monitored within normal ranges. Routine T2-weighted images were acquired using the Bruker:RARE sequence with the following parameters: repetition time = 4,926 ms, echo time = 33 ms, slice thickness = 0.65 mm, field of view = 35 × 35 mm, bandwidth = 152.587, image size = 256 × 256, flip angle = 90, and number of slices = 45. Resting-state blood oxygen level-dependent (BOLD) images were scanned using the Bruker:EPI sequence with the following parameters: repetition time = 2000 ms, echo time = 20 ms, slice thickness = 0.65 mm, field of view = 35 × 35 mm, bandwidth = 3,125, image size = 80 × 64, flip angle = 90, and repetitions = 400.

All fMRI data were analyzed using SPM12 and DPABI in MATLAB 2021. The paired *t* test was performed to determine the difference between rats before and after DRN DBS. In amplitude of low frequency fluctuations analysis, clusters that were significantly different were selected by setting *p* < 0.05 with a Gaussian random fields correction and clusters more than 129 voxels. In seed-based functional connectivity (FC) analysis, clusters that were significantly different were selected by setting *p* < 0.05 with Gaussian random fields correction and clusters of more than 264 voxels.

### Statistical analysis

Data are presented as the mean ± SEM, and the statistical analyses and plotting of the graphs were performed using GraphPad Prism 9 (GraphPad Software, California, USA).

## Results

### High-frequency, but not low-frequency, DBS of the DRN suppressed the reinstatement of methamphetamine-induced place preference

**Fig. 1:**
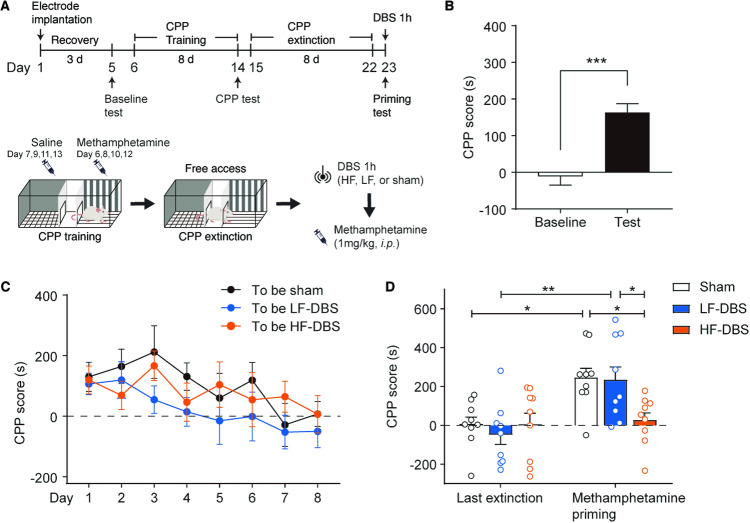
High-frequency (HF), but not low-frequency (LF), DBS of DRN suppressed drug-primed reinstatement of methamphetamine-induced CPP. **A** Top: Experimental timeline for the CPP-DBS procedure. Bottom: Diagram for the CPP and DBS processes. **B** CPP scores of the baseline preference and the expression test. Rats exhibited preference for the methamphetamine-paired side after CPP training. **C** The free access extinction sessions for the CPP-trained rats. The CPP scores of rats for the three to-be DBS groups returned to the baseline level in the last extinction session. **D** The methamphetamine-primed reinstatement for CPP. The CPP scores of HF-DBS group rats did not show a significant increase after drug priming compared with the two other groups and the last extinction test. *n* = 9/group, *p < 0.05; **p < 0.01; ***p < 0.001. Data are presented as the mean ± SEM.

First, we investigated the effect of different stimulus parameters for the DRN on the reinstatement of drug-induced seeking behavior. As shown in Fig. 1, rats underwent an 8-day methamphetamine CPP training, paired *t* test showed that rats had a strong preference for the methamphetamine-paired chamber (t_26_ = 4.937, *p* < 0.001). After that, the free access extinction procedure was performed until rats showed no preference to either side of the chamber (two-way ANOVA; extinction, F_5.210, 125.0_ = 3.871, *p* = 0.002). The rats were randomly divided into three groups: sham, high-frequency, and low-frequency DBS, and a total of 1 h of sham, high-frequency, or low-frequency electrical stimulation was delivered to the DRN, then the rats were given an injection of methamphetamine (1 mg/kg, *i.p*.) and performed the CPP test immediately. Two-way ANOVA showed that the CPP score of rats that underwent high-frequency DBS was not elevated after drug priming (DBS, F_2, 24_ = 2.719, *p* = 0.086; Test, F_1, 24_ = 13.54, *p* = 0.001; DBS × test interaction, F_2, 24_ = 2.702, *p* = 0.087; post hoc test, high-frequency DBS vs low-frequency DBS, *p* = 0.0303; high-frequency DBS vs sham DBS, *p* = 0.0207). These results suggest that high-frequency, but not low-frequency, DBS of DRN prevented the methamphetamine-primed reinstatement of place preference.

### High-frequency DBS of the DRN blocked methamphetamine-induced seeking behavior

**Fig. 2:**
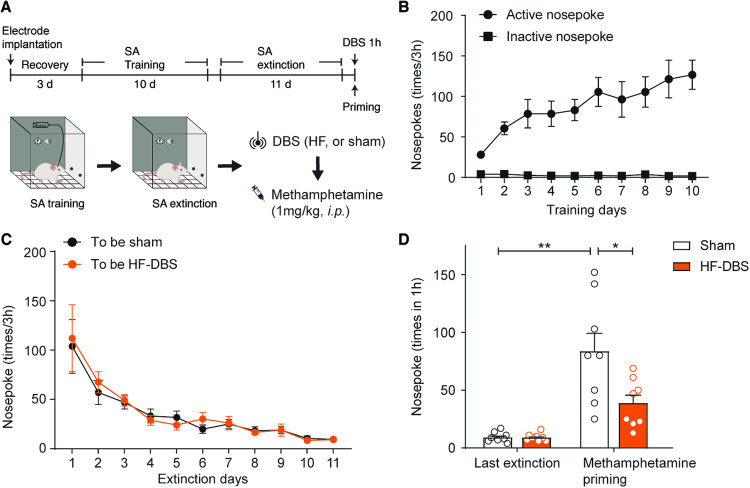
HF DBS of the DRN blocked methamphetamine-primed drug-seeking behavior. **A** Top: Experimental timeline for the methamphetamine self-administration (SA) DBS procedure. Bottom: Diagram for the SA and DBS processes. **B** Numbers of active and inactive nosepokes during SA training. **C** The free access extinction sessions for the SA-trained rats. The nosepoke of rats for the sham and HF DBS groups returned to the baseline level in the last extinction session. **D** The methamphetamine-primed reinstatement for SA. The number of nosepokes in HF DBS group rats did not show a significant increase after drug priming. *n* = 8/group, *p < 0.05; **p < 0.01. Data are presented as the mean ± SEM.

Compared to the traditional CPP paradigm, the self-administration model more accurately simulates voluntary drug intake and reinforcement, making it a superior approach for investigating craving and relapse [23, 24]. We then examined whether high-frequency DBS of the DRN could block methamphetamine-primed reinstatement in a self-administration model. As shown in Fig. 2, rats were trained to self-administer methamphetamine for 10 consecutive days to acquire stable self-administration behavior, and then they were given a 3 h extinction session per day until the nosepoke returned to baseline. Once the rats met the extinction criterion, they were divided into two groups: sham and high-frequency DBS. Then, a 1 h electrical stimulation was delivered to the DRN, after which a noncontingent methamphetamine injection (1 mg/kg, *i.p*.) was given. Two-way ANOVA showed that the counts of active nosepoke in methamphetamine-primed rats after high-frequency DBS were significantly lower than those in their controls, while methamphetamine-primed rats with sham DBS exhibited a significant elevation in the counts of active nosepoke (DBS, F_1, 28_ = 6.346, *p* = 0.018; priming, F_1, 28_ = 33.00, *p* < 0.001; DBS × priming interaction, F_1, 28_ = 6.346, *p* = 0.018; post hoc test, high-frequency DBS vs sham DBS, *p* = 0.007). These results indicated that high-frequency DBS of the DRN could block methamphetamine-primed seeking behaviors in rats.

### High-frequency DBS of the DRN did not affect responses to natural reward and locomotor activity or induce anxiety-like behavior in rats

**Fig. 3:**
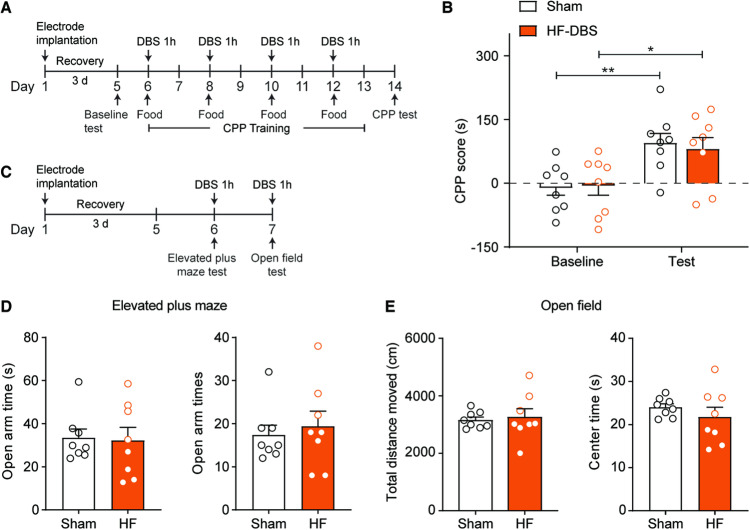
DBS affected neither responses to natural reward nor locomotor or anxiety-like behavior in rats. **A** Experimental timeline for the effect of DRN on the food CPP procedure. **B** CPP scores of the baseline preference and the expression test in the sham- and HF DBS rats. Both groups of rats exhibited a significant preference for the food-paired side after CPP training. **C** Experimental timeline for the elevated plus maze and open field tests. **D** The elevated plus maze tests. No difference in the total time and entries in the open arms was observed between the two groups of rats. **E** The open field tests. No difference in the total distance traveled or time spent in the central zone was observed between the two groups of rats. n = 8/group, *p < 0.05; **p < 0.01. Data are presented as the mean ± SEM.

Since high-frequency DBS of the DRN presents a potential therapy for methamphetamine-seeking behavior, we next assessed the safety and specificity of such manipulation. To examine whether high-frequency DBS of the DRN would affect responses to natural reward in rats, we performed high-frequency DBS of DRN before the food CPP conditioning. As shown in Fig. 3A, B, rats received a 1 h electrical stimulation before being placed into the food-paired chamber. Two-way ANOVA revealed that both sham and high-frequency DBS rats formed a significant preference for the food-paired side (DBS, F_1, 14_ = 0.026, *p* = 0.875; conditioning, F_1, 14_ = 22.51, *p* < 0.001; DBS × conditioning, F_1, 14_ = 0.211, *p* = 0.653). These results suggest that high-frequency DBS of the DRN had no impact on responses to natural reward in rats and may serve as a specifically target for methamphetamine relapse.

Subsequently, we determined whether high-frequency DBS of the DRN induced alterations in locomotor activity and anxiety-like behavior. As depicted in Fig. 3C–E, rats were subjected to the open field test and the elevated plus maze test. An unpaired *t* test showed that high-frequency and sham DBS groups had no difference in the open-arm time (t_14_ = 0.164, *p* = 0.872) and entries (t_14_ = 0.479, *p* = 0.639) in the elevated plus maze test. Additionally, no significant differences were observed in the distance traveled (t_14_ = 0.358, *p* = 0.726) and the time spent (t_14_ = 0.943, *p* = 0.362) in the central zone between the high-frequency and sham DBS groups in the open field test. Therefore, these data suggest that high-frequency DBS of the DRN neither affected locomotor activity nor induced anxiety-like behavior in rats.

### High-frequency DBS of the DRN modulated activity in brain regions related to drug addiction

**Fig. 4:**
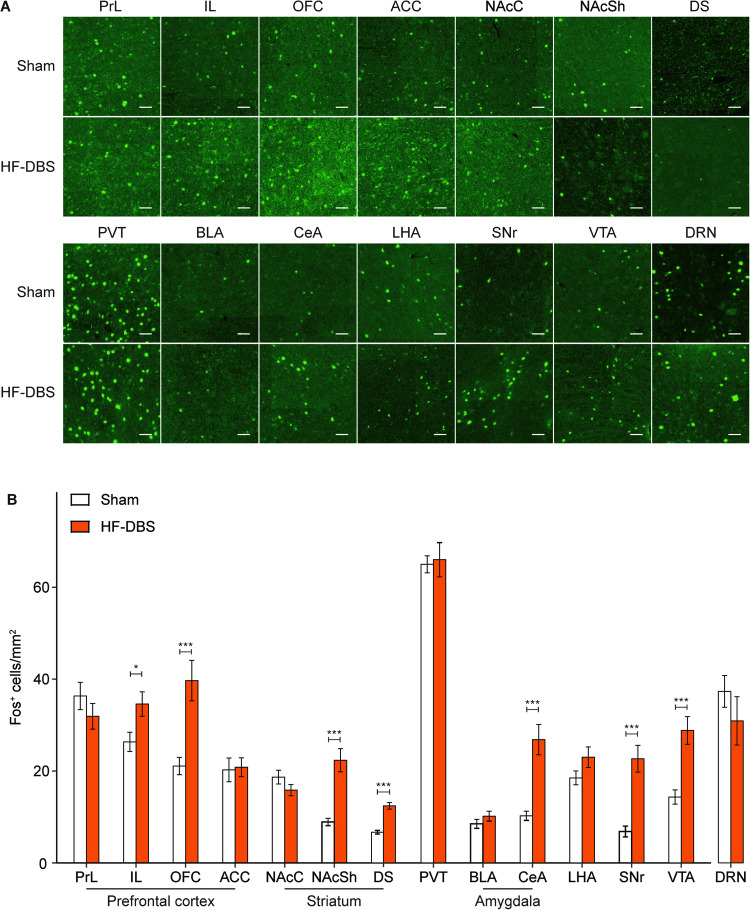
Brain regions that responded to HF DBS of the DRN. **A** Representative immunofluorescence images of c-Fos expression in the detected brain regions. Scale bar, 50 μm. PrL, prelimbic cortex; IL, infralimbic cortex; OFC, orbitofrontal cortex; ACC, anterior cingulate cortex; NAcC, nucleus accumbens core; NAcSh, nucleus accumbens shell; DS, dorsal striatum; PVT, paraventricular thalamus; BLA, basolateral amygdala; CeA, central amygdala; LHA, lateral hypothalamus area; SNr, substantia nigra pars reticulata; VTA, ventral tegmental area; DRN, dorsal raphe nucleus. **B** Quantity of c-Fos expression in rats in the sham- and HF-DBS groups. Twelve slices from 4 rats for each group, *p < 0.05; ***p < 0.001. Data are presented as the mean ± SEM.

To clarify the possible mechanism by which DRN DBS underlies the suppression of drug-primed reinstatement, we next detected alterations in neural activity by examining c-Fos expression in the brain regions involved in drug addiction. Rats that received 1 h high-frequency or sham DBS of the DRN were perfused 1.5 h later, and the counts of c-Fos number were compared between the high-frequency and sham DBS groups in each brain region. As shown in Fig. 4, an unpaired *t* test revealed that rats that underwent 1 h of DBS of the DRN showed significantly increased c-Fos-labeled neurons in the infralimbic cortex (IL, t_22_ = 2.43, *p* < 0.05), orbitofrontal cortex (OFC, t_22_ = 3.88, *p* < 0.001), NAc shell (t_22_ = 5.07, *p* < 0.001), dorsal striatum (DS, t_22_ = 6.98, *p* < 0.001), central amygdala (CeA, t_22_ = 4.82, *p* < 0.001), substantia nigra pars reticulata (SNr, t_22_ = 5.03, *p* < 0.001), and ventral tegmental area (VTA, t_22_ = 4.28, *p* < 0.001), which are closely related to addiction [2, 3, 25].

### High-frequency DBS of the DRN altered FC in brain regions associated with drug addiction

**Fig. 5:**
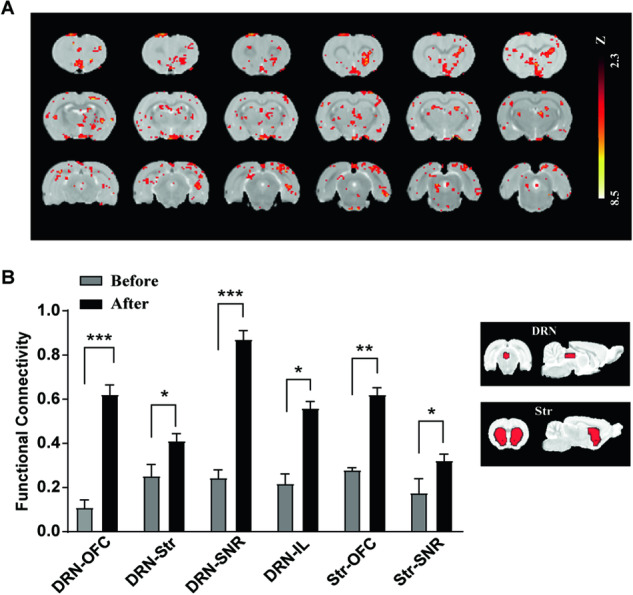
Amplitude of low-frequency fluctuation (ALFF) maps and functional connectivity after HF-DBS of the DRN in rats. **A** Images of brain slices showing regions with higher ALFF in rats after HF DBS of the DRN compared to before. **B** Functional connectivity between the selected regions of interest (ROIs) and addiction-related brain regions. A widespread increase in functional connectivity was observed after HF DBS of the DRN. *n* = 7, *p < 0.05; **p < 0.01; ***p < 0.001. Data are presented as the mean ± SEM.

Taking into consideration the disparities between ex vivo and in vivo research, we utilized fMRI to further investigate alterations in brain FC in rats following high-frequency DBS of the DRN. Representative blood-oxygen-level-dependent (BOLD) maps elicited by high-frequency DBS of the DRN are presented in Fig. 5A. Positive BOLD activations were predominantly observed in specific cortical areas, including the IL and OFC, as well as subcortical areas such as the striatum (Str), amygdala, insula, etc. (*p* < 0.05, corrected using Gaussian random field correction, one-sample *t* test). The BOLD signal exhibited a significant increase in the prelimbic cortex (PrL, 3.6 ± 0.13% change, *p* < 0.001), OFC (2.6 ± 0.04% change, *p* < 0.001), and IL (3.1 ± 0.07% change, *p* < 0.001) after high-frequency DBS of the DRN.

We then performed the FC analysis and selected DRN and Str as the seed region respectively. Rats that underwent high-frequency DBS of the DRN exhibited enhanced FC between the prefrontal cortex and brain regions, including the OFC, Str, and SNr, as presented in Fig. 5B. Specifically, a significant increase in the FC between the DRN and Str was observed, implying that high-frequency DBS treatment improved the strength of FC and facilitated the transmission of information within the dopaminergic circuit. Remarkable restorative effects on FC were observed when the Str was utilized as a seed region for high-frequency DBS treatment. Enhanced FC was observed between the OFC and SNr as well as the Str, suggesting a significant enhancement of the corticolimbic circuit subsequent to high-frequency DBS treatment.

## Discussion

Although several preclinical and clinical studies have shown that DBS has promising clinical applications for the treatment of addiction, it still faces a number of questions and challenges. To date, evidence for DBS in the treatment of addiction has predominantly focused on striatum as the stimulation target, with less research on other targets [7, 26–28]. As the key brain region of the reward system, NAc plays a crucial role in the initial stages of addiction, but with the progression of addiction, it is generally recognized that it goes beyond the dopamine reward system and that other brain regions are engaged [25, 29]. Meanwhile, due to the small sample sizes of participants, limitations of techniques and other factors, most of the studies have only carried out investigations on behaviors, leaving the underlying mechanisms to be elucidated. Moreover, research on DBS for substance addiction has also been mainly conducted on opioids and cocaine, as well as alcohol, whereas few studies have been reported on methamphetamine addiction [27, 28]. However, the abuse of amphetamine-type stimulants, mainly methamphetamine, has been increasing over the past decade and has become the second most abused drug [1]; thus, the development of new intervention targets and treatments is of great scientific and clinical importance. Here, we showed that the DRN could be a potential target in the use of DBS to prevent methamphetamine-seeking behaviors. We also explored the impact of DRN DBS on the activity of several brain regions and their FC in rats by both ex vivo and in vivo methods, which may be a potential mechanism for DRN in blocking methamphetamine relapse.

In the present study, we investigated the parameters and efficacy of DRN in the prevention of methamphetamine relapse and explored its possible mechanism. DRN is a brain region characterized by a heterogeneous intermingling of neurons, with varied functions being performed by distinct subtypes of neurons. Emerging evidence has proven that DRN engages in the expression of incentive memories, including memory in drug addiction [20, 30]. Moreover, the neuronal subtype of the DRN exhibits considerable heterogeneity, affording it the capacity to participate in a broad variety of behaviors. Specifically, dopaminergic neurons in the DRN play a distinct role in the expression of CPP, including food- and drug-induced CPP, while not contributing to the formation of CPP [31]. Furthermore, investigations have revealed that chronic methamphetamine self-administration results in activation of specific brain regions, including the DRN [32]. However, whether the DRN is implicated in the storage and expression of methamphetamine addictive memories remains unclear. Our data first, at least to our knowledge, demonstrated that high-frequency DBS of the DRN effectively inhibits both methamphetamine-primed reinstatement of place preference and drug seeking while not affecting responses to natural reward in rats. Moreover, high-frequency DBS of the DRN neither affected locomotor activity nor induced anxiety-like behavior. These results demonstrated that high-frequency DBS of the DRN may be a safe and effective therapeutic option for the treatment of relapse.

The repeated use of methamphetamine can induce enduring alterations in the brain reward systems and crucial regions involved in motivation, decision-making, and impulse control, which contribute to driving drug-seeking behaviors and relapse. Consequently, we conducted an investigation to examine the modifications in activity within various brain regions associated with drug addiction following high-frequency DBS of the DRN. Consistent with the results confirmed in previous studies that the medial prefrontal cortex, especially the IL, plays an important role in the extinction of drug addiction [33, 34], our data showed that the activity of the IL was significantly increased after high-frequency DBS of the DRN. Further studies using fMRI also found that high-frequency DBS of the DRN leads to increased FC between the DRN and IL. Moreover, studies have also shown that the NAc shell can be an effective target for addiction treatment, with high-frequency DBS of the NAc shell proving effective in inhibiting drug-primed seeking behavior for cocaine and methamphetamine [35, 36]. Meanwhile, we also observed that high-frequency DBS of the DRN led to activation in both the OFC and VTA. The OFC plays a fundamental role in motivated behavior, and deficits in its function elicit not only impulsive but also compulsive behaviors, which may underlie the mechanism of compulsive drug seeking and relapse [37, 38]. In line with our findings, high-frequency DBS of the OFC has also been shown to reduce the methamphetamine-induced reinstatement of seeking behaviors [39].

One limitation of this study is that we solely examined the effect of high-frequency DBS of the DRN on drug priming-induced reinstatement. However, relapse can be influenced by a variety of factors, including conditioned cues and stress, which were not evaluated in this study. Previous studies have shown that modulation of DRN activity exerts anxiolytic and antidepressant effects [12, 40]; therefore, we speculated that DRN may also suppress methamphetamine reinstatement triggered by conditioned cues and stress. Another limitation is that the present study did not fully explore the mechanism underlying the role of DBS in the treatment of methamphetamine relapse. We found that DBS of DRN did not cause changes in the c-Fos numbers in DRN brain regions. The mechanism of the effects of DBS is complex, and electrical stimulation not only affects neurons in target brain regions but also the passing by axons, axon terminals, and dendrites [5]. In addition, the neuronal types in the DRN brain area are also complex, and different neurons can form microcircuits that influence their activity. We speculated that DBS of DRN would not only cause changes in the activity of axon terminals that innervate the DRN, but also changes the activity of microcircuits in the DRN, and that these changes may contribute to the lack of difference in c-Fos activation in the DRN. Moreover, we also speculated that the c-Fos+ neurons activated in the high-frequency and sham DBS groups might be different types of neurons, and that the neurons activated in the high-frequency DBS group might be responsible for the altered activity in other brain regions. Previous studies have showed that both 5-HT and dopamine systems are extensively engaged in the formation of methamphetamine addiction and relapse [20, 41], but in this experiment, we did not explore the specific subtypes of neurons involved in the action of high-frequency DBS of the DRN. Moreover, we observed a significant increase in the FC between the DRN and Str; however, a comprehensive understanding of the specific patterns of this connectivity and its functional implications requires further investigation.

In conclusion, the present study demonstrated that DBS targeting the DRN prevented methamphetamine relapse and seeking in rats, which may be achieved by the recruitment of brain regions associated with addiction through the stimulation of afferent terminals. This study provides a novel and safe target, the DRN, that holds promise in reducing relapse and should be considered in circuit-specific investigations of DBS for drug addiction. Future studies could also combine DBS with techniques such as simultaneous electrophysiology recording to retrieve the characteristic pathobiological signals of drug addiction in real time and establish a novel intervention program for closed-loop DBS.

## Data Availability

The data that support the findings of this study are available from the corresponding author, Yu Shi, upon reasonable request.
